# Occurrence, Source Apportionment, and Risk Assessment of Antibiotics in the Zhuozhang River, China: A Specific Investigation in Water-Scarce and Human Activity-Intensive Regions

**DOI:** 10.3390/toxics13060422

**Published:** 2025-05-22

**Authors:** Juping Yan, Xiayang Wu, Ke Dong, Zhiyuan Zhang, Xuejun Sun, Shaopeng Gao, Jinxian Liu, Baofeng Chai

**Affiliations:** 1 School of Environmental and Resource, Taiyuan University of Science and Technology, Taiyuan 030024, China; 2 School of Environmental and Resource Sciences, Shanxi University, Taiyuan 030006, China; 3 Shanxi Key Laboratory of Ecological Restoration of the Loess Plateau, Shanxi University, Taiyuan 030006, China; 4 State Key Laboratory of Tibetan Plateau Earth System, Resources and Environment (TPESRE), Institute of Tibetan Plateau Research, Chinese Academy of Sciences, Beijing 100101, China; 5 Institute of the Loess Plateau, Shanxi University, Taiyuan 030006, China

**Keywords:** antibiotics, Zhuozhang River, source apportionment, risk assessment

## Abstract

Antibiotic contamination and its environmental impact in water-scarce and human activity-intensive regions have been poorly researched, particularly in the Zhuozhang River, China. Thus, this study investigated the occurrence, sources, and ecological risks of 27 different antibiotics in the Zhuozhang River, based on water samples collected from representative locations including major reservoirs, upstream of the river, the main river channel, and a wastewater treatment plant (WWTP). Results showed widespread contamination by quinolones, with concentrations ranged from 41.7 to 184.3 ng/L. Quinolones—particularly ofloxacin and cinoxacin—were identified as posing moderate ecological risks, with heightened concerns in the main river channel and wastewater treatment plant areas. Source apportionment using the positive matrix factorization model identified livestock farming as the dominant contributor to antibiotic pollution, accounting for 22.9% of the total antibiotic load in the river. These findings underscore the urgency of enhancing monitoring and management strategies to mitigate antibiotic contamination, especially in high-risk areas such as wastewater treatment plants and main river sections.

## 1. Introduction

Antibiotics are extensively employed in human medicine, veterinary practice, and aquaculture, primarily for the prophylaxis and treatment of microbial infections [1]. Recently, antibiotic contamination has emerged as a critical environmental issue of global concern [2,3], particularly due to its persistence in aquatic and terrestrial matrices and the associated ecotoxicological risks [1]. Escalating antibiotic consumption in low- and middle-income countries [4] has further intensified environmental loading and facilitated the propagation of antimicrobial-resistant determinants in natural ecosystems [5].

As one of the world’s largest producers and consumers of antibiotics [6], China manufactures ~210,000 tons of antibiotics annually [7,8]. Of these, around 180,000 tons are used in agriculture and human medicine [9]. Due to the relatively low metabolic degradation of antibiotics in humans and animals, an estimated 50–90% of administered antibiotics are excreted via urine and feces, releasing substantial quantities of these compounds into the environment in their parent forms or as bioactive metabolites [1,10,11]. In 2013, the total consumption of 36 frequently detected antibiotics in China reached 92,700 tons. Approximately 54,000 tons were excreted by humans and animals following administration, and an estimated 53,800 tons ultimately entered environmental compartments via treated wastewater discharge [8].

The concentrations and detection frequencies of antibiotics in Chinese rivers are generally higher than those reported in other countries [12,13]. The environmental fate of antibiotics in aquatic systems is constrained by their low biodegradability and strong sorptive behavior, which limit natural attenuation processes [14]. In groundwater, where microbial abundance is lower and photodegradation negligible [15], antibiotic degradation is further impeded, leading to their prolonged persistence. Additionally, antibiotics exhibit pseudo-persistence in environmental matrices [16], and chronic exposure may accelerate the dissemination of antibiotic-resistant genes [17], posing potential threats to ecosystem integrity and public health [18,19]. However, considerable knowledge gaps remain regarding the spatial distribution, primary sources, and ecological risks of antibiotics across different watersheds, particularly in regions with water scarcity and high levels of industrialization.

The Zhuozhang River is a major water system in Shanxi Province, located in the southwestern region of the Hai River Basin and spanning the provinces of Hebei, Henan, and Shanxi [20]. It flows through 13 counties, cities, and districts within Jinzhong and Changzhi. This watershed is among the most water-scarce regions in China while also serving as a crucial hub for industry, agriculture, energy production, and chemical manufacturing. Recently, population growth, urbanization, and rapid economic development have exacerbated water resource scarcity, while intensified industrial and agricultural activities may further deteriorate water quality. However, there is a lack of systematic research on the occurrence, sources, and ecological risks of antibiotic contamination in this watershed, and no relevant studies have been reported to date. Thus, this study represents the first investigation into antibiotic pollution in the Zhuozhang River Basin. It serves as a representative case reflecting pollution characteristics in water-scarce and human activity-intensive regions of China and has notable scientific and practical implications.

This study aims to provide a scientific basis for watershed management and antibiotic pollution control policies by (1) investigating the concentrations and spatial distribution of antibiotics in the Zhuozhang River; (2) identifying major antibiotic sources using the positive matrix factorization (PMF) model and correlation analysis; and (3) assessing the ecological risks associated with antibiotic contamination in an aquatic environment. The findings will enhance the understanding of antibiotic contamination patterns in the Zhuozhang River and provide essential data to support water environment protection and policy development in water-scarce regions.

## 2. Materials and Methods

### 2.1. Study Area

**Figure 1:**
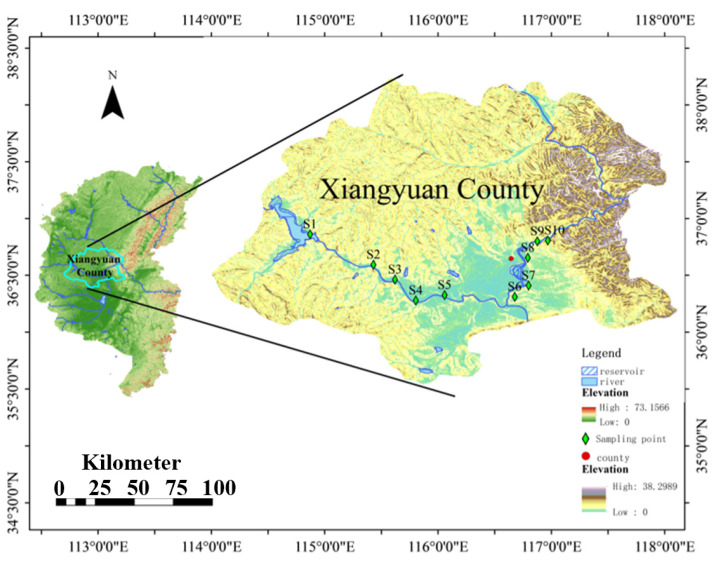
Sampling sites in the study area. Representative sampling sites were distributed along the river, namely major reservoirs (S1), the upstream of the river (UTR: S2, S3, S4), a WWTP (S5), and the main stream of the river (MSR: S6, S7, S8, S9, S10).

Zhuozhang River is an important water system in Shanxi Province and runs through two cities—Jinzhong and Changzhi—and thirteen counties. As the main tributary of the Zhang River, Zhuozhang River is in the southern portion of the Hai River Basin with a drainage area of 11,741 km^2^ [20]. With the rapid growth of urbanization and economic development, many industrial, agricultural, energy, and chemical bases have emerged along the river. Large amounts of domestic sewage and industrial and livestock-breeding wastewater enter the Zhuozhang River and generate considerable pollution. Thus, it is critical to investigate antibiotic occurrences in this river. As shown in Figure 1, representative sampling sites were distributed along the river, namely major reservoirs (S1), the upstream part of the river (UTR: S2, S3, S4), a WWTP (S5), and the main stream of the river (MSR: S6, S7, S8, S9, S10). All the samples were collected in August 2024.

### 2.2. Sample Extraction Method

The pretreatment steps were as follows: hydrophilic–lipophilic balance absorbent (Oasis-HLB, 200 mg, Waters, Milford, MA, USA), weak anion exchange adsorbent (Oasis-WAX, 100 mg, Waters, USA), and weak cation exchange reversed phase adsorbent (Oasis-WCX,100 mg, Waters, USA) were weighed and packed in the sampler. After shaking the sampler for 3 min to ensure the full mixing of the packing materials in the water, the samples were left to stand for 5 min to ensure the HLB and WAX fully adsorbed the antibiotics. Five milliliters of methanol (chromatogram purity, Thermo Fisher, Waltham, MA, USA) containing 0.1% ammonia and 5 mL methanol containing 1% formic acid (chromatogram purity, Dikma Technologies, Beijing, China) was used. The eluate was evaporated until dry under a gentle stream of high-purity nitrogen. The dried samples were redissolved in 1 mL methanol. After centrifuging at 10,000 rpm for 10 min, the supernatant was transferred to the sample vial. The volume of the water samples utilized for the extraction experiment was 500 mL. The equipment used was made of glass or stainless steel, immersed in 20% nitric acid for at least 24 h. Subsequently, the glasses were washed more than 10 times with pure water and baked at 320 °C for 4 h.

### 2.3. Detection Method

The antibiotic concentrations were detected using a high-performance liquid chromatography–mass spectrometer coupled to a Triple quadrupole mass spectrometer (LC-40D liquid chromatography SCIEX QTRAP^®^ 6500+ triple quadrupole mass spectrometer, Sciex, Framingham, MA, USA). Chromatographic separation was performed on a Waters ACQUITY UPLC^®^ BEH C18 column (2.1 mm × 100 mm, 1.7 μm). The mobile eluent was (a) ultrapure water containing 5 mmol/L ammonium acetate and (b) acetonitrile (*v*/*v*) containing 0.2% formic acid at a flow rate of 0.3 mL/min. The gradient elution was set as follows: start with 10% (b) from 0 to 2 min, increase to 97% within 3 min and hold for 2 min, then decrease to 10% (b) within 5.3 min and hold for 1.7 min until the column is equilibrated. The injection volume was 5 μL, and the column temperature was set at 40 °C. More detailed information on the detection method is shown in Text S1 and Figure S1.

### 2.4. Quality Assurance and Control

To ensure data accuracy, we carried out a labeled recovery experiment on the collected water samples. Internal antibiotic standards (100 ng) were added into the blank samples before pretreatment, and three groups of parallel samples were set up in each experiment. The recovery rate of 31 antibiotics was 73.12–115.3%, and the detection limit was 0.1–3.0 ng/L. The 31 antibiotics were as follows: enrofloxacin (ENR), nalidixic acid (NA), sparfloxacin (SPA), sulfadiazine (SDZ), sulfamethazine (SMZ), sulfamethoxazole (SMX), sulfaquinoxaline (SQX), sulfisoxazole (SSZ), sulfadoxine (SDM), oxolinicacid (OA), flumequin (FMQ), cinoxacin (CIN), sulfabenzamide (SB), sulfamethoxypyridazine (STD), sulfamonomethoxine (SMM), sulfaphenazole (SPP), lomefloxacin (LOM), ofloxacin (OFL), marbofloxacin (MAR), fleroxacin (FLX), sarafloxacin (SAL), orbifloxacin (OBI), difloxacin (DFL), oleandomycin (OLE), erythromycin (ERY), clarithromycin (CLR), roxithromycin (RTM), spiramycin (SPI), sulfathiazole (STZ), tylosin (TYL), and carbenicillin (CAR). Detailed information of all the chemicals, reagents, and materials used in this study are provided in the Supplemental Materials (Table S1), including their purity level and supplier information, as well as the recovery rates and the limit of quantification for these antibiotics.

### 2.5. Source Apportionment by the PMF Model

To quantify the contributions of different emission sources to antibiotics in the Zhuozhang River, the PMF receptor model was applied. The input data required by the PMF model were the concentration data for the antibiotics and the corresponding uncertainty data [21]. These values were determined as follows: (1) the concentration data below the detection limit were replaced with 1/2 of the detection limit and the corresponding uncertainty was calculated as 5/6 of the detection limit; and (2) for the concentration values beyond the detection limits, the uncertainties were calculated using the following equations [22,23]:

(1) $$\mathrm{If}\;X_{ij}\leq {3DL}_{i},\;\sigma_{ij}=\frac{{DL}_{i}}{3}+0.2\times C_{ij}$$

(2) $$\mathrm{If}\;X_{ij}>{3DL}_{i},\;\sigma_{ij}=\frac{{DL}_{i}}{3}+0.1\times C_{ij}$$

where $X_{ij}$ is the concentration value of the species *i* for the sample *j*, ${DL}_{i}$ is the detection limit of the species *i*, $\sigma_{ij}$ is the uncertainty value corresponding to the concentration $X_{ij}$, and $C_{ij}$ is the measured concentration.

Factor contributions and source profiles were derived using the PMF model when minimizing an objective function, *Q*, shown via the following equation:

(3) $$Q=\sum_{i=1}^{n}\sum_{j=1}^{m}{\left(e_{ij}/s_{ij}\right)}^{2}$$

where i is the time index; j is the variable index;$\;e_{ij}\;$ are the residuals; and $s_{ij}\;$ the error estimates of the data values. More information about the PMF model can be found in [24] and Zhang, et al. [25].

### 2.6. Risk Assessment of Antibiotics

The environmental risks of antibiotics in the aquatic environment were assessed based on the risk quotient (RQ), according to the European technical guidance document on risk assessment [26,27]. The RQ is estimated as the ratio between the measured environmental concentration (MEC) and predicted no-effect concentration (PNEC) [28]. The formula is expressed as follows:

(4) $${RQ}_{i}=\frac{{MEC}_{i}}{PNEC}$$

where ${MEC}_{i}$ is the concentration of antibiotic *i*. The PNEC was calculated using the following equations:

(5) $$PNEC={EC}_{50}({LC}_{50})/AF$$

(6) or PNEC=NOEC(LOEC)/AF

where LC_50_ or EC_50_ are short-term or acute toxicity data, NOEC or LOEC represent chronic toxicity, and AF is the assessment factor. The toxicity data of PNEC, EC_50_, NOEC, and AF values for the sensitive aquatic organisms are shown in Table S2.

## 3. Results and Discussion

### 3.1. Distribution Characteristics of Antibiotics in Surface Water

A total of 31 antibiotics were analyzed in the water samples along the Zhuozhang River. Of these antibiotics, 27 were detected, while SDZ, STZ, CAR, and TYL were not found at any site (Figure S2). The detected compounds included nine sulfonamides (SAs), five macrolides (MLs), and 13 quinolones (QNs). Notably, 10 QNs—including ENR, NA, and OFL—were detected at all sampling sites, with a 100% detection frequency (Figure S2). This indicates the widespread occurrence of QNs in the Zhuozhang River Basin and suggests that particular attention should be paid to their environmental presence.

The concentration levels of the three antibiotic classes in the Zhuozhang River are summarized in Table 1. QN concentrations ranged from 41.7 to 184.3 ng/L, with an average of 106.6 ng/L. The concentration ranges for MLs and SAs were 1.1 to 88.6 ng/L and 10.1 to 58.5 ng/L, with mean values of 32.3 and 20.8 ng/L, respectively. ML and SA concentrations were approximately one-third to one-fifth those of QNs. This phenomenon may be attributed to the primary sources of QNs, which are animal feces and human medical wastewater. Additionally, during the wet season, the enhanced adsorption of MLs onto river sediments—combined with increased river flow velocity—likely contributes to the lower ML and SA concentrations as SAs are hydrophilic [29,30]. Importantly, many high-concentration QNs such as OFL and ENR are also associated with elevated ecological risk values, as further elaborated in Section 3.3. Compared to other river basins, SA levels in the Zhuozhang River were higher than those reported in rivers in Australia and Spain, but considerably lower than those observed in the Liuxi River, Xiaoqing River, and Wenyu River Basin in China, and substantially lower than the levels detected in Vietnam (Table 1). In contrast, QNs in the Zhuozhang River exceeded those found in the Danjiangkou and Xiaoqing Rivers, the mainstream of the Songhua River, and Australian rivers, yet remained below the concentrations reported in Vietnam and India (Table 1). For MLs, the Zhuozhang River exhibited higher concentrations compared to the Danjiangkou Reservoir, the main stream of the Songhua River, and rivers in Australia, but lower concentrations than those detected in the Xiaoqing River and in Vietnamese rivers (Table 1). Collectively, SAs in the Zhuozhang River can be classified as representing low levels of pollution within a domestic context, whereas QNs and MLs represent relatively high pollution levels. On a global scale, all three antibiotic classes display moderate to high contamination levels, underscoring the necessity for continued monitoring and investigation of their spatial distribution and environmental behavior.

**Figure 2:**
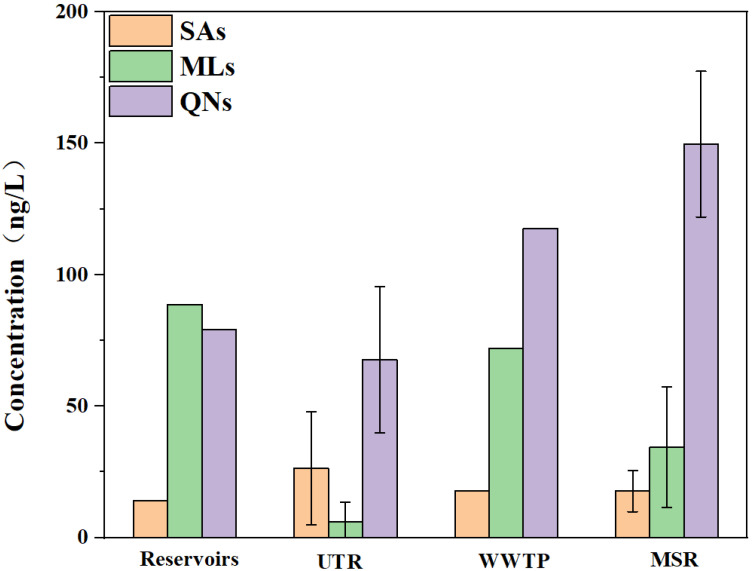
Concentration distribution characteristics of sulfonamides, macrolides, and quinolones in different sampling sites.

Antibiotic concentration profiles varied among the different functional zones (Figure 2). At the S1 site, the SA concentration was 14.1 ng/L, while the ML and QN concentrations were comparable at 88.6 and 79.2 ng/L, respectively. In the UTR, the average SA, ML, and QN concentrations were 26.4, 6.1, and 67.7 ng/L, respectively. At the WWTP, the SA, ML, and QN concentrations were 17.7, 71.8, and 117.7 ng/L, respectively. In the MSR, the average SA, ML, and QN concentrations were 17.7, 34.4, and 149.6 ng/L, respectively. It is noteworthy that, except for the UTR, all other sampling sites were dominated by MLs and QNs, with relatively low SA concentrations (Figure 2). In contrast, SA concentrations in the UTR were higher than that of MLs, likely attributed to the predominant use of SAs in poultry farming and livestock industries, which are more common in the villages near the UTR [29].

**Figure 3:**
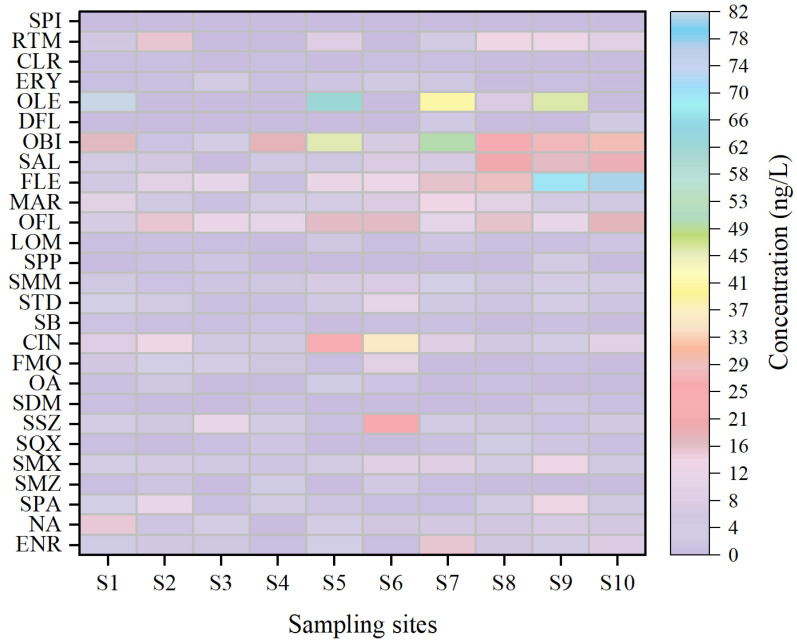
Spatial distribution of 27 antibiotics in the Zhuozhang River. Note: enrofloxacin (ENR), nalidixic acid (NA), sparfloxacin (SPA), sulfadiazine (SDZ), sulfamethazine (SMZ), sulfamethoxazole (SMX), sulfaquinoxaline (SQX), sulfisoxazole (SSZ), sulfadoxine (SDM), oxolinicacid (OA), flumequin (FMQ), cinoxacin (CIN), sulfabenzamide (SB), sulfamethoxypyridazine (STD), sulfamonomethoxine (SMM), sulfaphenazole (SPP), lomefloxacin (LOM), ofloxacin (OFL), marbofloxacin (MAR), fleroxacin (FLX), sarafloxacin (SAL), orbifloxacin (OBI), difloxacin (DFL), oleandomycin (OLE), erythromycin (ERY), clarithromycin (CLR), roxithromycin (RTM), spiramycin (SPI), sulfathiazole (STZ), tylosin (TYL), carbenicillin (CAR).

The concentration distribution of the 27 antibiotics at sampling sites S1–S10 is shown in Figure 3. Compared to other antibiotics, FLE consistently exhibited higher concentrations at each sampling site. Notably, at some sampling sites in the MSR (S9, S10), the concentrations reached their peak, with an accumulation of up to 247.6 ng/L across the entire basin. This is likely due to the broad-spectrum antimicrobial properties of FLE, widely used in human disease treatment and livestock farming, leading to an increasing concentration from the upstream to the main stream of the river [40]. At the major reservoir, the concentration of OLE was the highest among all sampling sites, reaching 82.1 ng/L, while the concentrations of other antibiotics were relatively low. In the UTR, the concentration variation patterns of antibiotics were similar to those observed at the major reservoir.

At the WWTP (S5), which directly or indirectly receives higher concentrations of antibiotic-contaminated wastewater, the SA concentration in the effluent was nearly zero, indicating the notable removal efficiency of sulfonamide by the treatment process [41]. Additionally, other compound concentrations were almost undetectable, except for SMM with concentrations that were 2–3 times higher (7.0 ng/L) than those at other sampling sites (Figure 3). Therefore, it can be inferred that SMM could potentially serve as a molecular marker for wastewater effluent in source apportionment studies. OLE (62.2 ng/L) and OBI (46.0 ng/L) concentrations at the WWTP were notably higher compared to other sampling sites (Figure 3). This could be attributed to the relatively low removal efficiency of these antibiotics by the wastewater treatment process [42].

In the MSR, FLE concentrations were particularly high, with an average concentration of 48.8 ng/L. The highest FLE concentration (81.1 ng/L) of all sampling sites was observed at S10 (Figure 3). Moreover, OBI, OLE, and other antibiotic concentrations in the MSR were higher than those in the UTR. This could be attributed to increased river flow and water volume during the wet season, as well as the influence of the nearby WWTP at S5, which contributed to the elevated antibiotic concentrations in the MSR [43]. Notably, several antibiotics—such as RTM, OFL, and CIN—that reached peak concentrations in the MSR and WWTP also exhibited high RQ values, particularly toward chronic toxicity endpoints for aquatic organisms like *Daphnia magna* (see Section 3.3). These overlaps between high concentrations and high-risk areas suggest that the spatial distribution of antibiotics is closely associated with their ecological impact.

### 3.2. Source Apportionment of Antibiotics

**Figure 4:**
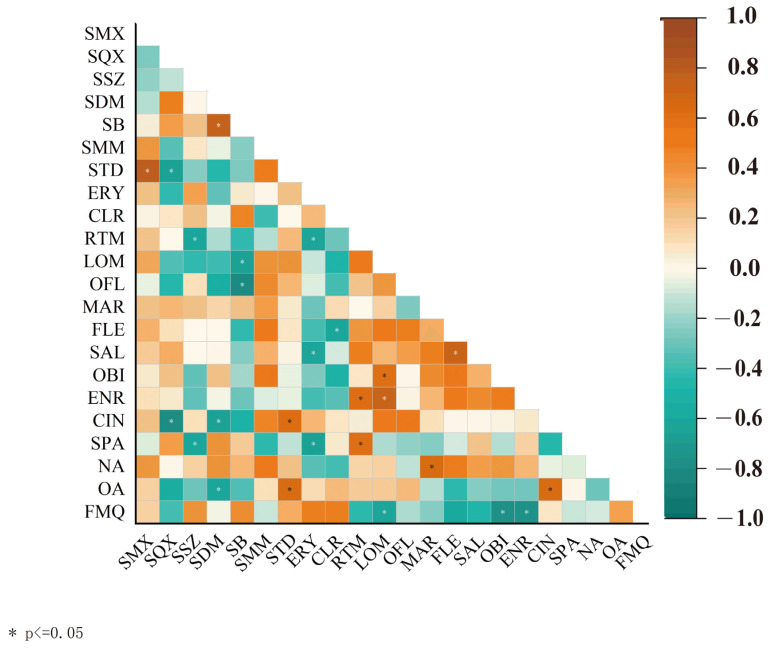
Correlation heatmap of antibiotic concentrations in the Zhuozhang River based on Spearman’s rank correlation coefficient analysis. Note: * represents *p* < 0.05.

To identify the source of antibiotics in the river, antibiotics with detection rates of >60% were chosen to be analyzed using Spearman’s correlation coefficient between different antibiotics (Figure 4). For the SAs, a significant positive correlation (*p* < 0.05) was exhibited between SMX and STD and SB and SDM, demonstrating that these antibiotics likely originate from similar pollution sources. For the MLs however, even for those antibiotics with similar chemical structures, there was no significant positive correlation between ERY, CLR, and RTM, indicating that their sources are likely different. For the QNs, a strong positive correlation (*p* < 0.05) was observed between LOM and ENR and OBI and MAR, demonstrating that these antibiotic sources are similar.

**Figure 5:**
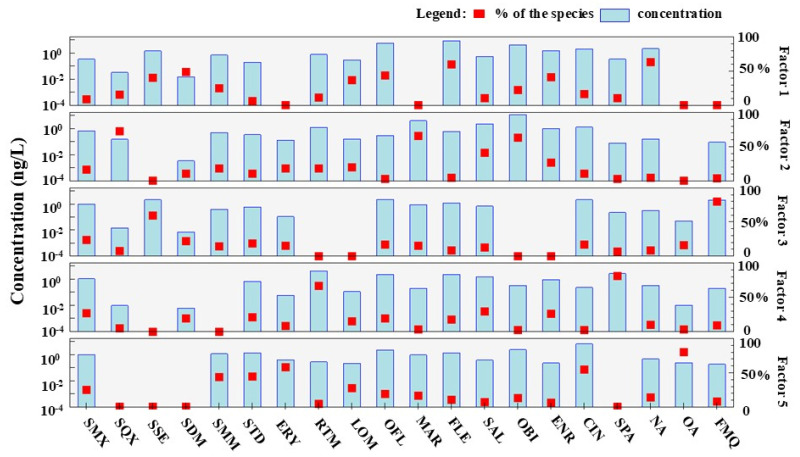
Antibiotic source profiles in the Zhuozhang River by PMF analysis.

To further quantify the primary origins of antibiotics in Zhuozhang River, the PMF receptor model was applied to the antibiotic concentrations. Five source contributors were included based on the PMF outputs (Figure 5). Factor 1 contributed more to OFL (43.5%) and ENR (41.2%) concentrations than other factors. OFL can reduce the incidence of disease and promote growth in livestock [21] and is the most commonly used QN drug in China, especially in poultry and swine breeding [44]. ENR is an antibiotic designated only for animals by the government [45,46]. Additionally, Factor 1 was dominated by high concentrations of NA (63.0%) and FLE (59.8%)—QNs used to treat different bacterial infections in animals. Therefore, Factor 1 is likely livestock discharge. Factor 2 was dominated by SQX (72.3%) and MAR (65.4%), which was identified as the source of farmland drainage. Cropland is the dominant land use, accounting for 41.38% of the total watershed area along the Zhang River [20]. Farmers in the Zhang Basin rely mainly on corn production [20], and fertilization is crucial for corn growth. Organic fertilizers containing SQX and MAR are often used by farmers, resulting in higher concentrations of these antibiotics in agricultural soil [47,48]. Additionally, rainfall and farmland irrigation leads to the spread of antibiotics from soils into rivers [49,50,51]. Thus, Factor 2 is likely farmland drainage.

Factor 3 was dominated by FMQ (79.8%) and SSZ (59.6%). The People’s Republic of China veterinary drug standard states that FMQ is a QN that can legally be used in aquaculture and has been widely used as a therapeutic tool to prevent bacterial diseases among aquaculture species [52]. Thus, Factor 3 is likely aquaculture. Factor 4 had higher RTM (66.5%) than the other factors (Figure 5). RTM is effective in treating infections of the respiratory tract [44] and is mainly released from hospital wastewater [42]. In addition, the levels of SMX concentrations in Factor 4 were higher than other factors. SMX is widely used to treat human urinary tract infections and is the dominate antibiotic in most sewage [44,53], especially in domestic sewage. Thus, Factor 4 is likely mixed pharmaceutical wastewater and domestic discharge.

In Factor 5, the primary antibiotic contributors were OA (80%) and ERY (58.5%). ERY is commonly found in WWTP effluent at mg/L levels [54] and are too recalcitrant to remove with conventional sewage treatments [54,55,56]. Thus, Factor 5 is represented by WWTP effluents. Moreover, as mentioned in Section 2.1, the results show that SMM is a biomarker of WWTP effluents compared with UTR and MSR. In the PMF analysis, Factor 5 higher SMM levels (43.6%) than other factors, which further confirm the results in Section 2.1.

**Figure 6:**
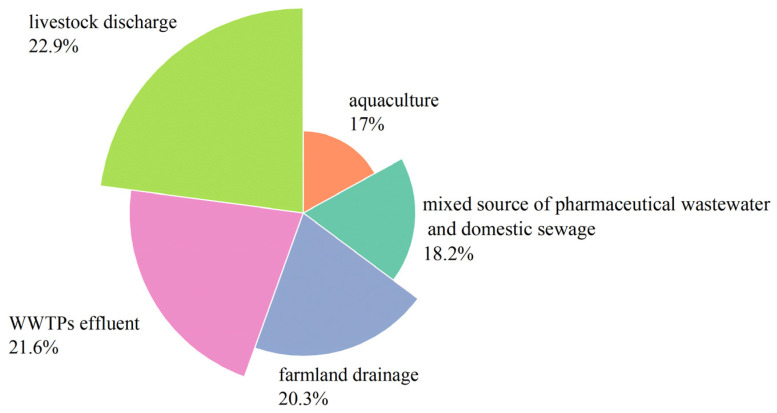
Contributions from five different sources to the total antibiotic amount in the Zhuozhang River.

Of all the sources, livestock discharge represents the largest proportion (22.9%) of antibiotics in the Zhuozhang River, followed by WWTP effluent (21.6%), farmland drainage (20.3%), pharmaceutical wastewater and domestic discharge (18.2%), and aquaculture (17.0%) (Figure 6). Substantial amounts (30–90%) of antibiotics administered to animals are excreted largely unmetabolized into waste streams via urine and feces [57], and wastewater discharged by livestock is almost directly discharged into rivers [21,58]. Thus, livestock discharge is the dominant antibiotic source in the Zhuozhang River.

### 3.3. Antibiotic Risk Assessments in the Zhuozhang River

**Figure 7:**
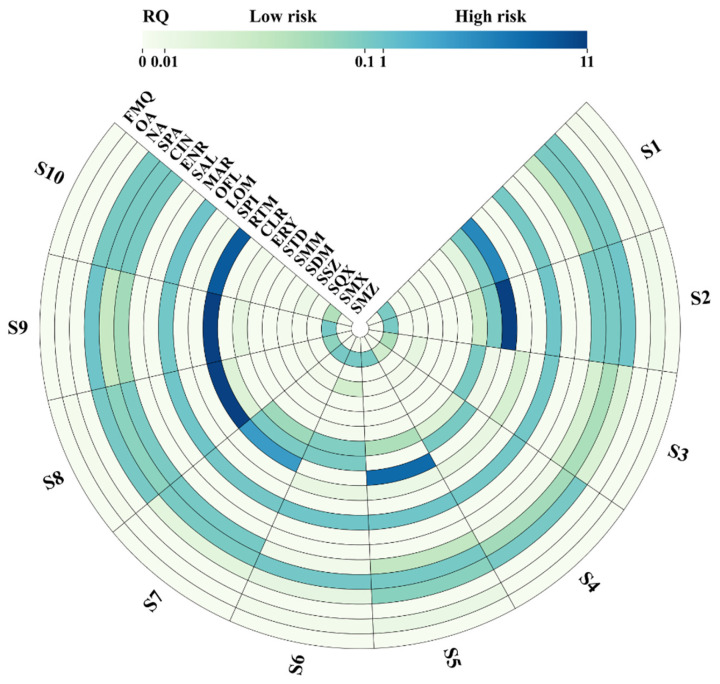
RQ values in different sampling sites.

This study employed the RQ method to comprehensively assess the ecological risks of 21 antibiotics in the Zhuozhang River during the high-flow period. The results are visually summarized in Figure 7, which displays the RQ values of each antibiotic across all sampling sites. Overall, 52% of the detected antibiotics exhibited an RQ value > 0.01 in at least one sampling site. The risk levels varied notably among the different antibiotic classes (Figure 7).

MLs demonstrated ecological risks at all sampling sites, with RQ values consistently >0.01. At certain locations, RQ values even surpassed 0.1, reaching a moderate-risk level. This suggests that MLs pose a widespread ecological risk in the Zhuozhang River Basin. Their extensive occurrence may be attributed to their low removal efficiency in wastewater treatment processes [59], leading to persistent accumulation in the aquatic environment and the formation of stable contamination throughout the watershed. Additionally, RTM, which exhibited the highest RQ value of the MLs, possesses potential bioaccumulation properties, which may further contribute to its elevated ecological risk [60].

Regarding ecological risk, QNs exhibited spatial heterogeneity. Of these, OFL, ENR, CIN, and SPA reached moderate-risk levels, while other QNs showed a relatively low risk. This spatial variation may be associated with point-source emissions and could also be influenced by dilution effects [26] and adsorption onto sediments [61], leading to lower aqueous-phase concentrations [59]. Additionally, most QNs undergo photodegradation and exhibit a high affinity for sludge, sediments, and soil [60,62,63]. Their strong adsorption onto suspended particles in river water further reduces their environmental risk [1,64]. The migration and distribution of QNs may vary considerably under different hydrological conditions, potentially resulting in localized accumulative ecological risks linked to point-source pollution. For high-risk areas, further research should involve dynamic monitoring during both high- and low-flow periods [26] to elucidate risk fluctuation patterns and provide data support for risk management. Among SAs, only SMX exhibited an RQ value > 0.1 at some sampling sites, reaching a moderate-risk level. This finding is consistent with studies carried out in the Yellow Sea [65], Bohai Sea [66], and Beibu Gulf of China [67]. Other SA RQ values remained <0.01, indicating negligible risk and suggesting that their overall environmental risk is relatively low. This could be attributed to the environmental degradation characteristics of SAs as they exhibit weak adsorption capacity and high hydrophilicity [68], making them more susceptible to dilution and dispersion through water flow [60]. However, antibiotics may still pose potential risks to non-target organisms [69]. Additionally, although individual SAs exhibit a low risk, their combined effects may enhance environmental hazards through synergistic pollution [70]. Therefore, further studies are needed to investigate the joint effects of SAs. The relatively higher risk level of SMX compared to other SAs may be related to its weak adsorption capacity or long half-life, resulting in a lower degradation rate and reduced dilution factor [71].

**Figure 8:**
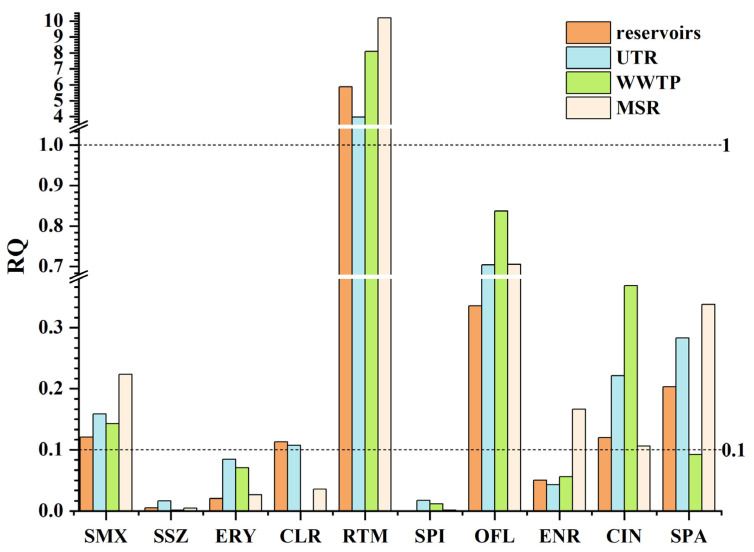
Region-based ecological risk assessment of selected high-risk antibiotics in the Zhuozhang River Basin.

Since not all antibiotics exhibited notable risks across all sites in Figure 7, Figure 8 further highlights those compounds with elevated RQ values in specific regions of the Zhuozhang River, grouped into four functional zones for focused analysis. In the reservoir, most antibiotics exhibited RQ values < 0.1, indicating relatively low overall ecological risk. However, SPA (RQ: 0.20) and OFL (RQ: 0.34) exceeded the low-risk threshold, suggesting localized ecological risks. These antibiotics may exhibit environmental persistence, allowing them to accumulate in the reservoir water. As a designated water source, reservoirs are typically subjected to strict water quality regulations, which help mitigate antibiotic pollution risks. Nevertheless, since reservoirs can receive pollution inputs from upstream, certain antibiotics may still persist in the aquatic environment. Moreover, reservoirs serve as key reserves of antimicrobial resistance [68], providing spatial conditions for the proliferation of resistant bacteria and their genetic material [72], which in turn influence the structure and function of microbial communities [73]. Additionally, reservoirs play a critical role in the formation and dissemination of resistance genes [74]. Therefore, to mitigate the emergence and spread of pathogenic antimicrobial resistant bacteria and reduce the risk of antibiotic resistance due to prolonged exposure to low antibiotic concentrations [75,76], the effective management and stringent monitoring of reservoirs are essential.

In the UTR, the ecological risk was relatively higher. Among these, SPA reached an exceptionally high RQ of 0.79 at S2, indicating near high-risk levels. OFL showed moderate risk at all UTR sites (RQ range: 0.58–0.79), reinforcing its persistence and ecological impact potential. Although RTM had the highest RQ value, indicating notable direct risks to aquatic ecosystems, OFL, due to its prolonged half-life in water—may pose more persistent ecological impacts [77]. Antibiotic pollution in this region is likely attributed to diffuse agricultural sources, household wastewater infiltration, and surface runoff. Additionally, direct discharge from poultry farming, meat processing, and aquaculture may serve as major pollution pathways, contributing to elevated antibiotic concentrations in river water [1].

At the WWTP, certain antibiotics exhibited higher ecological risk levels compared to the UTR. Notably, OFL had an RQ value of 0.84, falling into the moderate-risk category. CIN also reached a moderate-risk level with an RQ of 0.37. This pollution pattern suggests that WWTP exhibit limited removal efficiency for certain antibiotics, as some compounds are resistant to degradation or are poorly removed during treatment processes [1], leading to their accumulation in the receiving water bodies. Furthermore, WWTPs often receive antibiotic inputs from hospitals, livestock farming, and urban domestic wastewater [78], resulting in sustained high antibiotic concentrations even after treatment, ultimately increasing ecological risks through discharge into aquatic environments [57]. Thus, when conducting ecological risk assessments, the combined effects of antibiotics with other pollutants should also be considered [12]. Antibiotic degradation products may exhibit greater toxicity and environmental persistence than their parent compounds, necessitating the further evaluation of their potential risks [79].

In the MSR, OFL exhibited moderate ecological risk across all sites, with RQ values ranging from 0.53 to 0.88 and exceeding 0.8 at S6, S8, and S10, indicating persistent contamination. SPA demonstrated pronounced spatial variability, with RQs ranging from 0.02 (S6) to 0.9 (S9), suggesting site-specific influences and the potential for localized high-risk scenarios. CIN values varied between 0.05 and 0.53, occasionally exceeding the moderate-risk threshold. The observed heterogeneity may underscore the complex interplay of point and non-point sources, including effluent discharge, tributary inflows, and upstream contributions. These findings highlight the limitations of relying solely on average RQ values and emphasize the need for spatially resolved risk assessments to more accurately characterize ecological threats. Antibiotic contamination in the MSR is likely influenced by multiple factors, including WWTP discharges, upstream pollution inputs, tributary inflows, and hydrodynamic processes. Moreover, the physicochemical properties of antibiotics—such as solubility, adsorption potential, and photodegradation susceptibility—can affect their transport and degradation behavior in river systems [1], leading to the accumulation of certain antibiotics and consequently higher ecological risks in this region.

## 4. Conclusions

This study investigated the distribution and pollution sources of antibiotics and evaluated their ecological risks in the Zhuozhang River. The pollution levels of the three antibiotic classes in the Zhuozhang River are lower than those in Southeast Asian countries but fall within the moderate-to-high range in China. Five pollution sources were identified, and livestock farming and WWTP effluent were the most critical and the second most critical sources of antibiotics in the Zhuozhang River watershed, respectively. Furthermore, the RQ methodology revealed that antibiotics such as RTM, OFL, CIN, and SPA pose substantial ecological risks in multiple regions, particularly in the MSR and WWTP. These results emphasize the urgent need for targeted monitoring and management strategies, especially in ecologically sensitive areas. Collectively, this research provides a comprehensive understanding of antibiotic contamination sources and their ecological risks, offering valuable insights for the development of future environmental policies and management frameworks.

## Figures and Tables

**Table 1:** Comparison of SA, ML, and QN concentrations in rivers of the world.

River	ng/L	SAs	MLs	QNs	Reference
Zhuozhang River, China	Radius	10.1–58.5	1.1–88.6	41.7–184.3	This study
	Average	20.8	32.3	106.6	This study
Liuxi River, China	Radius	55.1–778.0	22.8–216.0	17.0–161.8	[31]
Danjiangkou, China	Radius	ND–149	ND–3.48	ND–9.17	[32]
Xiaoqing River, China	Average	81.64	138.8	86.97	[33]
The main stream of the Songhua River	Radius	ND-73.1	ND-11.5	ND-4.21	[34]
Wenyu River	Average	1046.7		400.4	[35]
Rivers in Southeast Queensland, Australia	Average	8	10	80	[36]
Ebro River Basin, Spain	Average	4.54	/	/	[37]
Musi River, India	Radius	/	/	35,420–6,278,000	[38]
Urban canals, Vietnam	Average	7940	6794	800	[39]

## Data Availability

The original contributions presented in this study are included in the article/Supplementary Materials. Further inquiries can be directed to the corresponding author(s).

## Supplementary Materials

The following supporting information can be downloaded at: https://www.mdpi.com/article/10.3390/toxics13060422/s1. Text S1: Validation of the detection methods; Figure S1: Chromatograms of internal standard and samples of three representative compounds of SAs, MLs, and QNs; Table S1: Detailed information of the antibiotics in this paper; Table S2: The PNEC, EC_50_, NOEC, and AF values were collected from previous studies to identify the sensitive aquatic organisms in this paper; Figure S2: The detection rate of 31 antibiotics in the Zhuozhang River [1]. References [80,81,82,83,84,85,86,87,88,89,90,91,92,93,94,95,96,97] are cited in the Supplementary Materials.
