# Supplementary material for: Occurrence, Source Apportionment, and Risk Assessment of Antibiotics in the Zhuozhang River, China: A Specific Investigation in Water-Scarce and Human Activity-Intensive Regions
Source: Toxics. 2025 May 22;13(6):422. doi: 10.3390/toxics13060422 (PMC12196876; doi:10.3390/toxics13060422)
Supplement: Supplementary file 1 [file toxics-13-00422-s001.zip › toxics-3633797-supplementary.pdf]

# Occurrence, Source Apportionment, and Risk Assessment of Antibiotics in the Zhuozhang River, China: A Specific Investigation in Water-Scarce and Human Activity-Intensive Regions

Juping Yan [a], Xiayang Wu [a], Ke Dong [a], Zhiyuan Zhang [a], Xuejun Sun [b,e,*], Shaopeng Gao [c], Jinxian Liu [b,d,e], Baofeng Chai [b,d,e]

[a] School of Environmental and Resource, Taiyuan University of Science and Technology, Taiyuan 030024, China

[b] School of Environmental and Resource Sciences, Shanxi University, Taiyuan 030006, China

[c] State Key Laboratory of Tibetan Plateau Earth System, Resources and Environment (TPESRE), Institute of Tibetan Plateau Research, Chinese Academy of Sciences, Beijing, 100101, China

[d] Institute of the Loess Plateau, Shanxi University, Taiyuan, 030006, China

[e] Key Laboratory of Ecological Restoration of the Loess Plateau in Shanxi Province, Shanxi University, Taiyuan, 030006, China

* Corresponding author.

(Xuejun Sun)

E-mail: sunxuejun@sxu.edu.cn

Tel.: +86-0351-7010600

Fax: +86-0351-7010600

# Contents


Text S1. Validation of the detection methods........ 3

Figure S1. Chromatograms of internal standard and samples of three representative compounds of SAs, MLs, and QNs........ 4

Table S1. Detailed information of the antibiotics in this paper........ 5

Table S2. The PNEC, EC50, NOEC, and AF values were collected from previous studies to identify the sensitive aquatic organisms in this paper........ 8

Figure S2. The detection rate of 31 antibiotics in the Zhuozhang River........ 13

Text S1. Validation of the detection methods.

The chromatographic method used in this study followed the method of Lu (2024). This method was developed and validated in a previously published study. According to the results of the validation experiment in the study, as cited in the References, the concentrations of the target pollutants in water bodies did not affect the treatment efficacy of the method, as demonstrated by investigating the recovery rates at low (1 ng/L), medium (5 ng/L), and high (10 ng/L) concentrations of antibiotics. The average recovery of antibiotics at low (1 ng/L), medium (5 ng/L), and high (10 ng/L) concentrations was 95 ± 38%, 82 ± 34%, and 90 ± 20%, respectively. The result of the validation experiment demonstrates applicability across different concentrations of water samples.

Reference

Lu Zijun, High-throughput screening of new pollutants in water based on dispersive solid-phase extraction [D], Research Center for Eco-Environmental Sciences, Chinese Academy of Sciences (P17-P30).

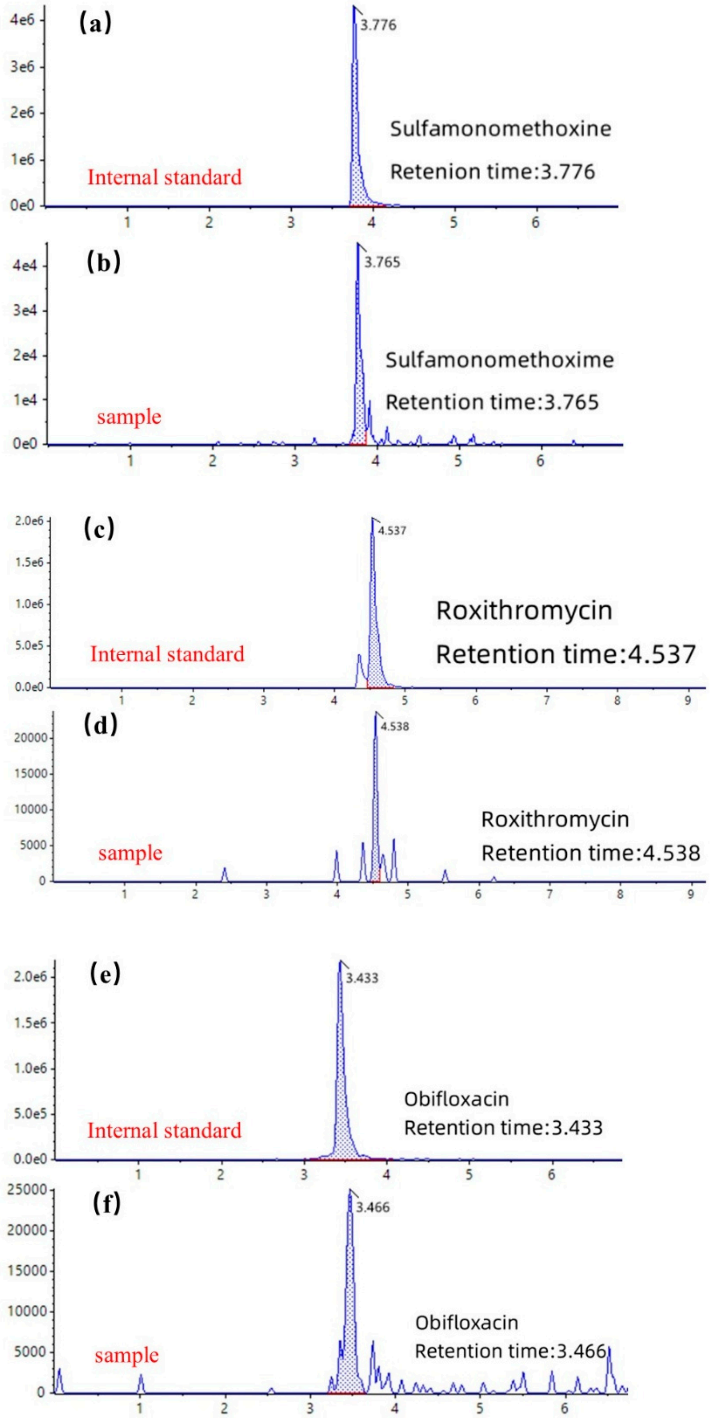


Figure S1. Chromatograms of internal standard and samples of three representative compounds of SAs, MLs, and QNs.

Table S1. Detailed information of the antibiotics in this paper.

| Antibiotics | abbreviation | formula | CAS | Purity | Production Company | Recovery (%) | Limit of quantification (ng/L) | Limit of Detection (ng/L) | Q1 Mass | Q3 Mass | Retention time |
|---|---|---|---|---|---|---|---|---|---|---|---|
| Enrofloxacin | ENR | $C_{19}H_{22}FN_3O_3$ | 93106-60-6 | 98.0% | Alta Scientific Co., Ltd | 80.9 | 0.3 | 0.1 | 360 | 316.1 | 3.98 |
| | | | | | | | | | 360 | 245.1 | |
| Nalidixic acid | NA | $C_{12}H_{12}N_2O_3$ | 389-08-2 | 99.9% | Alta Scientific Co., Ltd | 69.1 | 0.3 | 0.1 | 233 | 215 | 4.41 |
| | | | | | | | | | 233 | 187 | |
| Sparfloxacin | SPA | $C_{19}H_{22}F_2N_4O_3$ | 111542-93-9 | 99.9% | Alta Scientific Co., Ltd | 68.2 | 0.3 | 0.1 | 393 | 349.2 | 4.04 |
| | | | | | | | | | 393 | 292.2 | |
| Sulfadiazine | SDZ | $C_{10}H_{10}N_4O_2S$ | 68-35-9 | 98.0% | Alta Scientific Co., Ltd | 123 | 0.6 | 0.2 | 250.4 | 156 | 2.65 |
| | | | | | | | | | 250.4 | 184.2 | |
| Sulfamethazine | SMZ | C12H14N4O2S | 57-68-1 | 98.0% | Alta Scientific Co., Ltd | 104 | 0.3 | 0.1 | 279.1 | 186.1 | 4.03 |
| | | | | | | | | | 279.1 | 156 | |
| Sulfamethoxazole | SMX | $C_{10}H_{11}N_3O_3S$ | 723-46-6 | 98.0% | Alta Scientific Co., Ltd | 94.3 | 0.06 | 0.02 | 254.2 | 156 | 4.21 |
| | | | | | | | | | 254.2 | 92.1 | |
| Sulfaquinoxaline | SQX | $C_{14}H_{12}N_4O_2S$ | 59-40-5 | 98.0% | Alta Scientific Co., Ltd | 106 | 0.03 | 0.01 | 301.1 | 156 | 4.33 |
| | | | | | | | | | 301.1 | 108 | |
| Sulfisoxazole | SSZ | $C_{11}H_{13}N_3O_3S$ | 127-69-5 | 98.7% | Alta Scientific Co., Ltd | 64.4 | 0.3 | 0.1 | 268 | 156 | 3.98 |
| | | | | | | | | | 268 | 113 | |
| Sulfadoxine | SDM | $C_{12}H_{14}N_4O_4S$ | 2447-57-6 | 98.4% | Alta Scientific Co., Ltd | 88.3 | 0.06 | 0.02 | 311.1 | 156.1 | 4.34 |
| | | | | | | | | | 311.1 | 108.2 | |
| Oxolinic acid | OA | $C_{13}H_{11}NO_5$ | 14698-29-4 | 95.0% | Alta Scientific Co., Ltd | 122 | 0.06 | 0.02 | 262 | 244.1 | 4.52 |
| | | | | | | | | | 262 | 216.1 | |
| Flumequin | FMQ | $C_{14}H_{12}FNO_3$ | 42835-25-6 | 98.9% | Alta Scientific | 130 | 0.3 | 0.1 | 262.1 | 244.1 | 4.51 |

| | | | | | | | | | | | |
|---|---|---|---|---|---|---|---|---|---|---|---|
| | | | | | Co., Ltd | | | | 262.1 | 202.1 | |
| Cinoxacin | CIN | C12H10N2O5 | 28657-80-9 | 99.9% | Alta Scientific Co., Ltd | 83.4 | 0.06 | 0.02 | 263.1 | 217.1 | 4.19 |
| | | | | | | | | | 263.1 | 245 | |
| Sulfabenzamide | SB | C13H12N2O3S | 127-71-9 | 98.0% | Alta Scientific Co., Ltd | 68.2 | 0.21 | 0.07 | 277.1 | 156 | 3.98 |
| | | | | | | | | | 277.1 | 108.2 | |
| Sulfamethoxypyridazine | STD | C11H12N4O3S | 80-35-3 | 99.0% | Alta Scientific Co., Ltd | 103 | 0.3 | 0.1 | 281 | 156 | 4.04 |
| | | | | | | | | | 281 | 126.1 | |
| Sulfamonomethoxine | SMM | $C_{11}H_{12}N_4O_3S$ | 1220-83-3 | 98.0% | Alta Scientific Co., Ltd | 107 | 0.3 | 0.1 | 281.1 | 156 | 4.04 |
| | | | | | | | | | 281.1 | 126.1 | |
| Sulfaphenazole | SPP | $C_{15}H_{14}N_4O_2S$ | 526-08-9 | 99.8% | Alta Scientific Co., Ltd | 124 | 0.3 | 0.1 | 315 | 156 | 4.36 |
| | | | | | | | | | 315 | 108 | |
| Lomefloxacin | LOM | $C_{17}H_{19}F_2N_3O_3$ | 98079-51-7 | 99.0% | Alta Scientific Co., Ltd | 59.6 | 0.6 | 0.2 | 352.3 | 265.2 | 3.95 |
| | | | | | | | | | 352.3 | 308.3 | |
| Ofloxacin | OFL | $C_{18}H_{20}FN_3O_4$ | 82419-36-1 | 98.0% | Alta Scientific Co., Ltd | 69.8 | 0.6 | 0.2 | 362.2 | 318.1 | 3.91 |
| | | | | | | | | | 362.2 | 261.1 | |
| Marbofloxacin | MAR | C17H18FNO3S | 873945-29-0 | 99.0% | Alta Scientific Co., Ltd | 77.8 | 0.6 | 0.2 | 363.1 | 320 | 3.87 |
| | | | | | | | | | 363.1 | 345.1 | |
| Fleroxacin | FLX | $C_{17}H_{18}F_3N_3O_3$ | 79660-72-3 | 98.0% | Alta Scientific Co., Ltd | 73.6 | 0.15 | 0.05 | 370 | 326.1 | 3.27 |
| | | | | | | | | | 370 | 269.2 | |
| Sarafloxacin | SAL | $C_{20}H_{17}F_2N_3O_3$ | 98105-99-8 | 98.0% | Alta Scientific Co., Ltd | 85.7 | 1.5 | 0.5 | 386.1 | 341.8 | 4.04 |
| | | | | | | | | | 386.1 | 298.8 | |
| Orbifloxacin | OBI | $C_{19}H_{20}F_3N_3O_3$ | 113617-63-3 | 99.9% | Alta Scientific Co., Ltd | 97 | 0.3 | 0.1 | 396.2 | 352.1 | 3.43 |
| | | | | | | | | | 396.2 | 295 | |
| Difloxacin | DFL | $C_{21}H_{19}F_2N_3O_3$ | 98106-17-3 | 99.5% | Alta Scientific Co., Ltd | 80.4 | 0.45 | 0.15 | 400.1 | 355.7 | 4.05 |
| | | | | | | | | | 400.1 | 298.9 | |
| Oleandomycin | OLE | C35H61NO12 | 3922-90-5 | 98.2% | Alta Scientific Co., Ltd | 121 | 0.03 | 0.02 | 688.4 | 158.2 | 4.24 |
| | | | | | | | | | 688.4 | 544.3 | |
| Erythromycin | ERY | $C_{37}H_{67}NO_{13}$ | 114-07-8 | 98.0% | Alta Scientific Co., Ltd | 100 | 0.3 | 0.1 | 734.4 | 576.2 | 4.11 |
| | | | | | | | | | 734.4 | 158.1 | |

| | | | | | | | | | | | |
|---|---|---|---|---|---|---|---|---|---|---|---|
| Clarithromycin | CLR | $C_{38}H_{69}NO_{13}$ | 81103-11-9 | 99.0% | Alta Scientific Co., Ltd | 105 | 0.3 | 0.1 | 748.7<br>748.7 | 590.4<br>158.3 | 4.44 |
| Roxithromycin | RTM | $C_{41}H_{76}N_2O_{15}$ | 80214-83-1 | 98.0% | Alta Scientific Co., Ltd | 98.2 | 0.06 | 0.02 | 837.6<br>837.6 | 679.5<br>158.1 | 4.54 |
| Spiramycin | SPI | $C_{43}H_{74}N_2O_{14}$ | 8025-81-8 | 96.9% | Alta Scientific Co., Ltd | 88.7 | 0.3 | 0.1 | 843.4<br>843.4 | 174.1<br>318.3 | 4.01 |
| Sulfathiazole | STZ | $C_9H_9N_3O_2S_2$ | 72-14-0 | 98.0% | Alta Scientific Co., Ltd | 105 | 0.3 | 0.1 | 256<br>256 | 156<br>108 | 4.01 |
| Tylosin | TYL | $C_{46}H_{77}NO_{17}$ | 1401-69-0 | 99.0% | Alta Scientific Co., Ltd | 92.6 | 0.3 | 0.1 | 916.6<br>916.6 | 174<br>772.5 | 4.28 |
| Carbenicillin | CAR | $C_{17}H_{18}N_2O_6S$ | 4697-36-3 | 98.0% | Alta Scientific Co., Ltd | 75.1 | 0.6 | 0.2 | 411.1<br>411.1 | 160<br>217.1 | 4.2 |

Table S2. The PNEC, EC50, NOEC and AF values were collected from previous studies to identify the sensitive aquatic organisms in this paper.

| Antibiotics | Non-target organism | Toxicity data ($mg\cdot L^{-1}$) | AF | PNEC ($ng\cdot L^{-1}$) | Reference |
|---|---|---|---|---|---|
| SDZ | M. aeruginosa | $EC_{50} = 0.135$ | 1000 | 135 | [1] |
| SMZ | Lemna minor | $EC_{50} = 1.277$ | 1000 | 1277 | [2] |
| SMX | S. leopoliensis | $EC_{50} = 0.03$ | 1000 | 30 | [3] |
| SQX | Daphnia magna | $EC_{50} = 131$ | 1000 | 131000 | [4] |
| SSZ | Lemna minor | $EC_{50} = 0.62$ | 1000 | 620 | [2] |
| SDM | Lemna gibba | $EC_{50} = 0.445$ | 1000 | 445 | [5] |
| SMM | Chlorella vulgaris | $EC_{50} = 5.9$ | 1000 | 5900 | [6] |
| STZ | Daphnia magna | $EC_{50} = 8.2$ | 1000 | 8200 | [7] |
| STD | Lemna minor | $EC_{50} = 3.82$ | 1000 | 3820 | [8] |
| ERY | Anabaena sp. | $EC_{50} = 0.022$ | 1000 | 22 | [9] |
| CLR | P. subcapitata | $EC_{50} = 0.002$ | 1000 | 2 | [10] |
| RTM | P. subcapitata | NOEC = 0.01 | 100 | 1 | [11] |

| | | | | | |
|---|---|---|---|---|---|
| SPI | M.aeruginosa | $EC_{50} = 0.005$ | 1000 | 5 | [12] |
| LOM | Fish | $EC_{50} = 770$ | 1000 | 770000 | [13] |
| OFL | M.aeruginosa | $EC_{50} = 0.02$ | 1000 | 20 | [14] |
| MAR | C.dubia | $EC_{50} = 17$ | 1000 | 17000 | [15] |
| SAL | Fish | $EC_{50} = 190$ | 1000 | 190000 | [13] |
| ENR | M.aeruginosa | $EC_{50} = 0.05$ | 1000 | 50 | [14] |
| CIN | Lemna minor | $EC_{50} = 0.0671$ | 1000 | 67.1 | [16] |
| SPA | M.aeruginosa | $EC_{50} = 0.015$ | 1000 | 15 | [12] |
| NA | V. fischeri | $EC_{50} = 199.94$ | 1000 | 199940 | [17] |
| OA | M.aeruginosa | $EC_{50} = 0.18$ | 1000 | 180 | [12] |
| FMQ | P. subcapitata | $EC_{50} = 9.3$ | 1000 | 9300 | [18] |

## References


1. Lützhoft, H.C.H.; Halling-Sorensen, B.; Jorgensen, S.E. Algal toxicity of antibacterial agents applied in Danish fish farming. *Archives of Environmental Contamination and Toxicology* **1999**, *36*, 1-6, doi:10.1007/s002449900435.
2. Bialk-Bielinska, A.; Stolte, S.; Arning, J.; Uebers, U.; Boeschen, A.; Stepnowski, P.; Matzke, M. Ecotoxicity evaluation of selected sulfonamides. *Chemosphere* **2011**, *85*, 928-933, doi:10.1016/j.chemosphere.2011.06.058.
3. Ferrari, B.; Mons, R.; Vollat, B.; Fraysse, B.; Paxéus, N.; Lo Giudice, R.; Pollio, A.; Garric, J. Environmental risk assessment of six human pharmaceuticals: Are the current environmental risk assessment procedures sufficient for the protection of the aquatic environment? *Environmental Toxicology and Chemistry* **2004**, *23*, 1344-1354, doi:10.1897/03-246.
4. De Liguoro, M.; Fioretto, B.; Poltronieri, C.; Gallina, G. The toxicity of sulfamethazine to *Daphnia magna* and its additivity to other veterinary sulfonamides and trimethoprim. *Chemosphere* **2009**, *75*, 1519-1524, doi:10.1016/j.chemosphere.2009.02.002.
5. Brain, R.A.; Johnson, D.J.; Richards, S.M.; Sanderson, H.; Sibley, P.K.; Solomon, K.R. Effects of 25 pharmaceutical compounds to *Lemna gibba* using a seven-day static-renewal test. *Environmental Toxicology and Chemistry* **2004**, *23*, 371-382, doi:10.1897/02-576.
6. Yang, Q.; Gao, Y.; Ke, J.; Show, P.L.; Ge, Y.; Liu, Y.; Guo, R.; Chen, J. Antibiotics: An overview on the environmental occurrence, toxicity, degradation, and removal methods. *Bioengineered* **2021**, *12*, 7376-7416, doi:10.1080/21655979.2021.1974657.
7. Jung, J.; Kim, Y.; Kim, J.; Jeong, D.-H.; Choi, K. Environmental levels of ultraviolet light potentiate the toxicity of sulfonamide antibiotics in *Daphnia magna*. *Ecotoxicology* **2008**, *17*, 37-45, doi:10.1007/s10646-007-0174-9.

8. Pro, J.; Ortiz, J.A.; Boleas, S.; Fernández, C.; Carbonell, G.; Tarazona, J.V. Effect assessment of antimicrobial pharmaceuticals on the aquatic plant *Lemna minor*. *Bulletin of Environmental Contamination and Toxicology* **2003**, *70*, 290-295, doi:10.1007/s00128-002-0208-1.
9. Yan, Z.; Yang, H.; Dong, H.; Ma, B.; Sun, H.; Pan, T.; Jiang, R.; Zhou, R.; Shen, J.; Liu, J.; et al. Occurrence and ecological risk assessment of organic micropollutants in the lower reaches of the Yangtze River, China: A case study of water diversion. *Environmental Pollution* **2018**, *239*, 223-232, doi:10.1016/j.envpol.2018.04.023.
10. Isidori, M.; Lavorgna, M.; Nardelli, A.; Pascarella, L.; Parrella, A. Toxic and genotoxic evaluation of six antibiotics on non-target organisms. *Science of the Total Environment* **2005**, *346*, 87-98, doi:10.1016/j.scitotenv.2004.11.017.
11. Yang, L.-H.; Ying, G.-G.; Su, H.-C.; Stauber, J.L.; Adams, M.S.; Binet, M.T. Growth-inhibiting effects of 12 antibacterial agents and their mixtures on the freshwater microalga *Pseudokirchneriella subcapitata*. *Environmental Toxicology and Chemistry* **2008**, *27*, 1201-1208, doi:10.1897/07-471.1.
12. Halling-Sorensen, B. Algal toxicity of antibacterial agents used in intensive farming. *Chemosphere* **2000**, *40*, 731-739, doi:10.1016/s0045-6535(99)00445-2.
13. Xu, M.; Huang, H.; Li, N.; Li, F.; Wang, D.; Luo, Q. Occurrence and ecological risk of pharmaceuticals and personal care products (PPCPs) and pesticides in typical surface watersheds, China. *Ecotoxicology and Environmental Safety* **2019**, *175*, 289-298, doi:10.1016/j.ecoenv.2019.01.131.

14. Robinson, A.A.; Belden, J.B.; Lydy, M.J. Toxicity of fluoroquinolone antibiotics to aquatic organisms. *Environmental Toxicology and Chemistry* **2005**, *24*, 423-430, doi:10.1897/04-210r.1.

15. Kergaravat, S.V.; Hernandez, S.R.; Maria Gagneten, A. Second-, third- and fourth-generation quinolones: Ecotoxicity effects on *Daphnia* and *Ceriodaphnia* species. *Chemosphere* **2021**, *262*, doi:10.1016/j.chemosphere.2020.127823.

16. Li, M.; Wei, D.; Du, Y. Acute toxicity evaluation for quinolone antibiotics and their chlorination disinfection processes. *Journal of Environmental Sciences* **2014**, *26*, 1837-1842, doi:10.1016/j.jes.2014.06.023.

17. Backhaus, T.; Scholze, M.; Grimme, L.H. The single substance and mixture toxicity of quinolones to the bioluminescent bacterium *Vibrio fischeri*. *Aquatic Toxicology* **2000**, *49*, 49-61, doi:10.1016/s0166-445x(99)00069-7.

18. Munch Christensen, A.; Ingerslev, F.; Baun, A. Ecotoxicity of mixtures of antibiotics used in aquacultures. *Environmental Toxicology and Chemistry* **2006**, *25*, 2208-2215, doi:10.1897/05-415r.1.

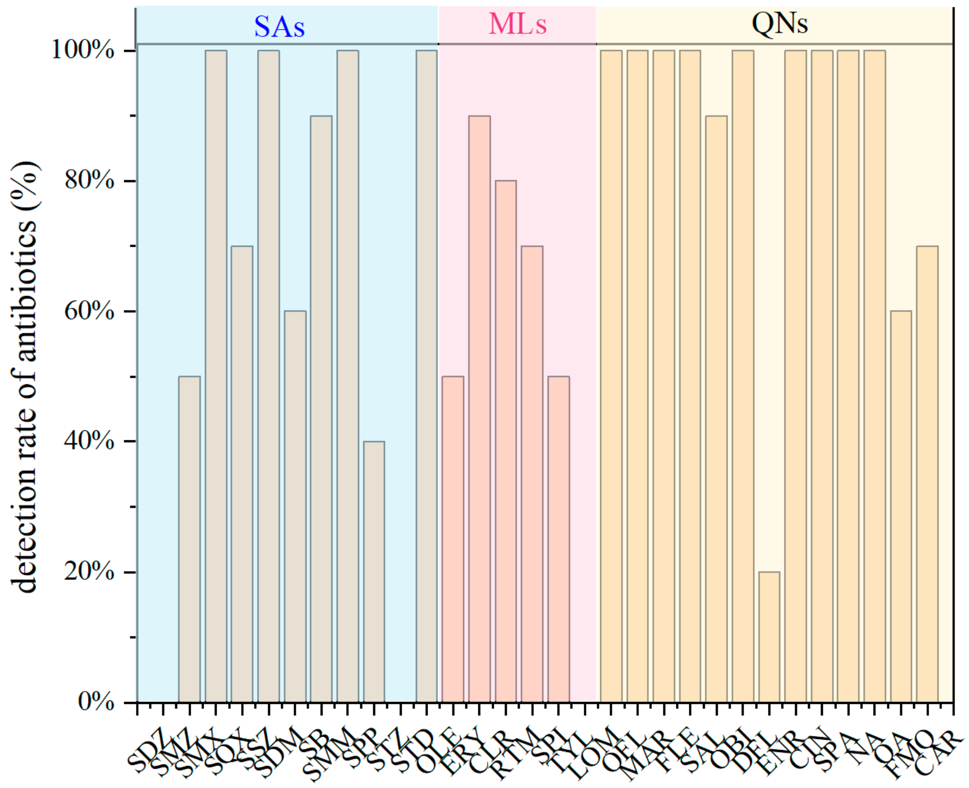


Figure S2. The detection rate of 31 antibiotics in the Zhuozhang River.
